# Synchronous ileo-ileal and ileocolic intussusception caused by an inverted giant Meckel’s diverticulum in an adult: a rare case report

**DOI:** 10.1097/RC9.0000000000000527

**Published:** 2026-05-12

**Authors:** Mohamed Sayed, Abdulhakim Bin Onayq, Muhammad Nassr, Abdelfattah Ayoub, Muhammad Abukhater

**Affiliations:** a Sulaiman AlRajhi University, College of Medicine, Department of Clinical Sciences, AlBukayriyah, Qassim, Saudi Arabia; b King Fahad Medical City, Surgical Oncology Department, Minimally Invasive and Bariatric Surgery Unit, Riyadh, Saudi Arabia; c King Saud University, College of Medicine, Riyadh, Saudi Arabia

**Keywords:** adult intussusception, ileocolic intussusception, ileo-ileal intussusception, Meckel’s diverticulum, small bowel obstruction, case report

## Abstract

**Introduction:**

Adult intussusception is a rare clinical entity, typically associated with a pathological lead point. Meckel’s diverticulum, although the most common congenital gastrointestinal anomaly, seldom presents with complications in adulthood. Synchronous intussusception involving multiple bowel segments is exceptionally uncommon.

**Presentation of case:**

A 21-year-old man presented with a 2-week history of intermittent colicky abdominal pain, abdominal distension, bilious vomiting, and constipation. Imaging revealed features of both ileo-ileal and ileocolic intussusception with small bowel obstruction. Diagnostic laparoscopy confirmed dual intussusceptions with viable bowel. Following reduction, an inverted giant Meckel’s diverticulum was identified as the lead point. Segmental small bowel resection with primary anastomosis was performed. Histopathology demonstrated diverticulitis with ectopic gastric mucosa and no malignancy. The postoperative course was uneventful.

**Discussion:**

In adults, intussusception is usually secondary to an identifiable lesion, often requiring surgical management. Meckel’s diverticulum may act as a lead point, particularly when inverted, mimicking an intraluminal mass. Preoperative diagnosis is challenging due to nonspecific symptoms; however, computed tomography plays a crucial role in detection and surgical planning. Synchronous intussusception in adults is extremely rare and typically underreported.

**Conclusion:**

This case highlights a rare presentation of synchronous ileo-ileal and ileocolic intussusception caused by an inverted giant Meckel’s diverticulum. Early recognition and prompt surgical intervention are essential to prevent complications and ensure favorable outcomes.

## Introduction

Intussusception occurs when a segment of bowel telescopes into an adjacent distal segment, leading to luminal obstruction and, in some cases, impairment of the mesenteric blood supply^[^1^]^. Failure to promptly recognize the condition may lead to persistent venous congestion, which can subsequently progress to ischemia, bowel necrosis, and ultimately perforation. While intussusception is a well-recognized cause of intestinal obstruction in children, it is distinctly uncommon in adults and accounts for only a small proportion of bowel obstruction cases in this population^[^2^]^. The underlying cause of intussusception differs markedly between age groups. In children, the condition is often idiopathic, whereas in adults, it is usually secondary to a pathological lead point^[^2^]^. Structural lesions, including benign and malignant tumors, are frequently implicated, which explains why surgical management is generally favored in adult patients^[^1^]^.

HIGHLIGHTS

- Adult intussusception is rare and usually has a pathological lead point.
- Meckel’s diverticulum can cause small bowel intussusception in adults.
- We report synchronous ileo-ileal and ileocolic intussusception.
- Giant inverted Meckel’s diverticulum was the underlying lead point.
- Computed tomography imaging enabled diagnosis, and surgery led to a successful outcome.

Meckel’s diverticulum is the most common congenital anomaly of the gastrointestinal tract and arises from the incomplete involution of the omphalomesenteric duct during embryonic development^[^3^]^. It is a true diverticulum originating from the antimesenteric border of the distal ileum and is present in approximately 2% of the general population^[^3^]^. Although most individuals remain asymptomatic, and complications most often present during childhood, Meckel’s diverticulum may lead to inflammation, hemorrhage, intestinal obstruction, perforation, and intussusception^[^4^]^. The presence of ectopic mucosa, most commonly gastric in origin, can further contribute to mucosal ulceration and gastrointestinal bleeding^[^5^]^. When clinically significant disease occurs in adults, symptoms are often nonspecific, and establishing a preoperative diagnosis can be difficult^[^6–8^]^.

The mechanism by which Meckel’s diverticulum causes intussusception varies. It may act as a fixed lead point due to inflammation, fibrotic thickening, or inversion into the intestinal lumen^[^7,8^]^. Inverted Meckel’s diverticulum has been described as an effective lead point because it behaves as an intraluminal mass, resulting in progressive invagination during peristalsis^[^8^]^. Synchronous intussusception involving both the small and large bowel in adults is exceedingly rare, particularly when caused by Meckel’s diverticulum acting as a pathological lead point^[^7,8^]^.

The present case describes an unusual presentation of a giant Meckel’s diverticulum, resulting in concurrent ileo-ileal and ileocolic intussusception. This contributes to the limited literature on synchronous adult intussusception and highlights the potential for a single congenital anomaly to produce complex and atypical presentations.

## Methods

This work has been reported in line with the SCARE criteria^[^9^]^.

## Presentation of the case

A 21-year-old Saudi male presented to the emergency department after experiencing intermittent central colicky abdominal pain and nausea for 2 weeks, followed by multiple episodes of bilious vomiting over the last few days. He also reported constipation and abdominal distension for 1 week. He reported previous similar episodes that were less severe and had been managed as irritable bowel symptoms. There was no history of fever, chills, or gastrointestinal bleeding. The patient was medically free, with no previous surgeries or inherited digestive disorders within close relatives.

On arrival, the patient was hemodynamically stable, with a blood pressure of 121/69 mmHg, a heart rate of 90 beats per minute, a respiratory rate of 20 breaths per minute, a temperature of 36.6°C, and an oxygen saturation of 96% on room air. Abdominal examination revealed mild distension with localized tenderness over the right lower quadrant and suprapubic region, accompanied by guarding. Digital rectal examination demonstrated hard stool without blood or palpable masses. The remainder of the physical examination was unremarkable.

**Figure 1.**
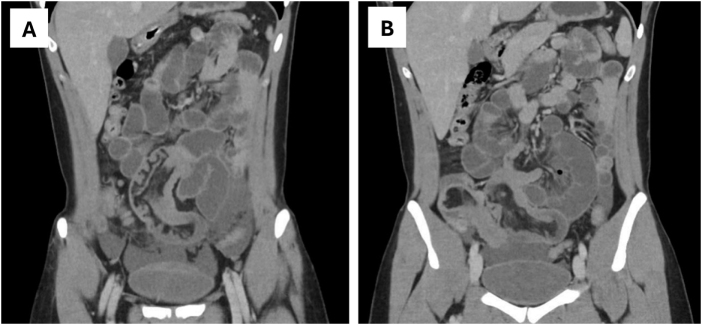
(A) Contrast-enhanced computed tomography showing dilated small bowel loops with a characteristic “bowel-within-bowel” configuration in the mid-abdomen, consistent with ileo-ileal intussusception. (B) Contrast-enhanced computed tomography demonstrating ileocolic intussusception with associated mild wall thickening of the distal ileum.

Initial laboratory investigations demonstrated a white blood cell count of 8.5 × 10^9^/L (reference range: 4.5–11 × 10^9^/L), hemoglobin level of 16.3 g/dL (reference range: 13.5–17.5 g/dL), and hematocrit of 49% (reference range: 41–53%). Plain abdominal radiography showed multiple air–fluid levels consistent with small bowel obstruction, without evidence of free intraperitoneal air. Contrast-enhanced computed tomography (CT) of the abdomen and pelvis revealed dilated small bowel loops measuring up to 3.2 cm in diameter, with a collapsed colon. A transition point was identified in the mid-abdomen, demonstrating a characteristic “bowel-within-bowel” appearance consistent with ileo-ileal intussusception (Fig. 1A). A second intussusception was identified more distally at the ileocolic junction, associated with mild bowel wall thickening (Fig. 1B).

Based on the clinical presentation and radiologic findings, the decision was made to proceed with urgent surgical intervention. The patient received preoperative prophylactic antibiotics, and diagnostic laparoscopy was performed. Intraoperatively, the bowel appeared viable with no evidence of ischemia, although significant small bowel dilatation was observed. Intussusception of the terminal ileum into the cecum was identified, along with a synchronous ileo-ileal intussusception. Laparoscopic reduction was successfully achieved. Following reduction, an abnormal segment of bowel was noted, characterized by focal indentation and the presence of a mobile, firm intraluminal mass within the previously invaginated ileal segment, raising concern for an underlying pathological lead point.

**Figure 2.**
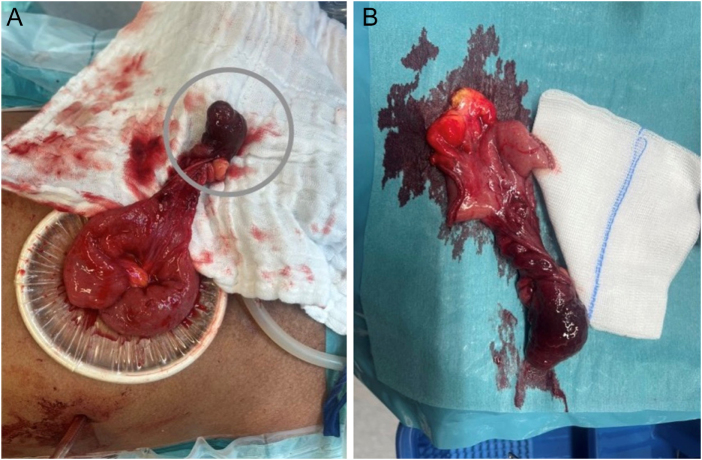
(A) Intraoperative photograph of the exteriorized small bowel revealing an invaginated Meckel’s diverticulum (gray circle), acting as a pathological lead point for ileo-ileal intussusception. (B) Gross specimen of the resected small bowel showing a giant Meckel’s diverticulum with a broad base.

A small midline incision measuring approximately 2 cm was made to exteriorize the affected bowel segment for further evaluation (Fig. 2A). Intraoperative inspection revealed a Meckel’s diverticulum with a broad base that had inverted into the bowel lumen and was identified as the lead point for the ileo-ileal intussusception. Segmental resection of the involved small bowel, including the Meckel’s diverticulum, was performed (Fig. 2B). A side-to-side functional end-to-end small bowel anastomosis was constructed using a 60-mm gastrointestinal anastomosis stapler with a blue cartridge. The common enterotomy was closed using a transverse anastomosis stapler, and the staple line was reinforced with interrupted 3-0 polyglactin (Vicryl) sutures. Hemostasis was meticulously ensured, and closed-suction drains were placed in the pelvis and along the right paracolic gutter, extending to the subhepatic region.

Gross examination of the resected specimen revealed a Meckel’s diverticulum measuring 7.5 cm in length, consistent with a giant Meckel’s diverticulum. Histopathological evaluation demonstrated features of diverticulitis, including ischemic changes, mucosal ulceration, fibrinopurulent exudate, hemorrhage, and ectatic vessels. Ectopic gastric mucosa and Brunner’s glands were identified within the diverticulum. There was no histological evidence of dysplasia or malignancy.

Seven days after surgery, the patient was discharged from the hospital in stable condition. The postoperative course was uneventful, and the patient remained asymptomatic at a 3-week follow-up.

## Discussion

Intussusception in adults is an uncommon clinical entity and differs fundamentally from pediatric intussusception in terms of etiology, presentation, and management. In contrast to children, in whom intussusception is often idiopathic, adult cases are usually secondary to a definable pathological lead point. Consequently, adult intussusception frequently presents as bowel obstruction and typically requires operative management, both for definitive diagnosis and treatment^[^1^]^.

Meckel’s diverticulum represents a rare but recognized cause of adult intussusception. Although it is the most common congenital anomaly of the gastrointestinal tract, with an estimated prevalence of approximately 2% in the general population, the majority of individuals with Meckel’s diverticulum remain asymptomatic throughout life^[^3^]^. When complications occur, they most often present during childhood. Symptomatic Meckel’s diverticulum in adults is uncommon and may manifest as inflammation, bleeding, obstruction, or intussusception^[^4^]^. The nonspecific nature of symptoms often results in delayed or missed preoperative diagnosis^[^6–8^]^. Meckel’s diverticulum may cause intussusception through inflammation, fibrotic thickening, or inversion into the intestinal lumen^[^7,8^]^. Inverted Meckel’s diverticulum is a particularly effective lead point because it behaves as an intraluminal mass and promotes progressive invagination during peristalsis^[^8^]^. In the present case, the diverticulum was broad-based and inverted, which likely contributed to the development of ileo-ileal intussusception.

CT plays a central role in the evaluation of suspected adult intussusception^[^10^]^. Typical findings include a bowel-within-bowel configuration, mesenteric fat and vessels within the intussuscepted segment, and proximal bowel dilatation^[^10^]^. In adults, CT imaging not only confirms the diagnosis but also assists in identifying potential lead points and assessing bowel viability^[^10^]^. In the present case, CT imaging was instrumental in identifying both intussusceptions preoperatively, allowing for timely surgical intervention.

Surgical management remains the standard of care for adult intussusception because of the high likelihood of an underlying pathological lesion^[^1^]^. In adult patients with bowel obstruction secondary to structural pathology, including intussusception, laparoscopy is increasingly employed as both a diagnostic and therapeutic modality in hemodynamically stable individuals. It allows direct visualization, facilitates reduction, and enables targeted resection when a pathological lead point is identified, while offering the benefits of minimally invasive surgery. Current practice supports a laparoscopic-first approach in selected cases managed by experienced surgeons, with a low threshold for conversion to open surgery in the presence of marked bowel distension, suspected ischemia, or technical limitations^[^2^]^. In this patient, diagnostic laparoscopy allowed reduction and resection of the affected segment, including the diverticulum. Although reduction may be considered in selected cases with viable bowel, resection of the involved segment is recommended when a lead point is identified to prevent recurrence and to allow histopathological evaluation^[^11^]^. Segmental small bowel resection with primary anastomosis is widely accepted as the preferred approach in cases involving Meckel’s diverticulum^[^11^]^.

Histopathological examination in this case demonstrated diverticulitis with ischemic changes and the presence of ectopic gastric mucosa. These findings support the role of chronic inflammation and mucosal heterotopia in contributing to the diverticulum’s pathological behavior^[^5^]^. Importantly, no evidence of dysplasia or malignancy was identified, which is consistent with most reported cases^[^12–14^]^ but does not diminish the necessity for resection given the potential for serious complications.

Synchronous intussusceptions involving more than one segment of the bowel are exceedingly rare, particularly in adults. The literature contains only a limited number of reports describing double or simultaneous intussusceptions, most of which occur in pediatric patients and are associated with multiple lead points^[^15–17^]^. Several reports have described adult intussusception secondary to Meckel’s diverticulum presenting with intestinal obstruction, including cases reported by Bouassida *et al*^[^18^]^, Marascia *et al*^[^19^]^, Zorn *et al*^[^20^]^, and Liu *et al*^[^21^]^, all of which required operative confirmation and segmental resection. Schaedlich *et al* similarly reported that abdominal pain and vomiting were the most common presenting symptoms in patients older than 15 years, with surgical resection being the definitive treatment when a pathological lead point is identified^[^13^]^.

Most described adult cases involve a single segment of bowel. In contrast, the coexistence of ileo-ileal and ileocolic intussusception caused by a single pathological lead point, as observed in this patient, represents an extremely uncommon presentation and adds to the limited literature on synchronous adult intussusception.

## Conclusion

Adult intussusception is an uncommon cause of bowel obstruction and is usually related to an underlying pathological lead point. Although Meckel’s diverticulum is typically asymptomatic and more often identified in childhood, it should be considered in young adults presenting with features of intestinal obstruction. This case describes a rare presentation of synchronous ileo-ileal and ileocolic intussusception caused by a giant Meckel’s diverticulum. Contrast-enhanced CT is central to diagnosis and operative planning, and definitive treatment requires surgical exploration with resection of the affected bowel segment.

## Data Availability

All data relevant to this case report are included within the article. Additional information is available from the corresponding author upon reasonable request.
